# Prosthetic rehabilitation with fixed prosthesis of a 5-year-old child with Hypohidrotic Ectodermal Dysplasia and Oligodontia: a case report

**DOI:** 10.1186/s13256-019-2268-4

**Published:** 2019-11-08

**Authors:** Reema AlNuaimi, Mohammad Mansoor

**Affiliations:** Dubai Health Authority, Dubai, United Arab Emirates

**Keywords:** Ectodermal dysplasia, Pediatric dentistry, Fixed prosthesis, Prosthetic rehabilitation, Prosthodontics, Oligodontia, Children with special needs, Growth and development

## Abstract

**Background:**

Ectodermal dysplasia is a rare genetic disorder that affects ectodermally derived structures, including teeth, nails, hair, and sweat glands. Hypohidrotic ectodermal dysplasia is the most common type, with oligodontia being the most striking dental feature. Prosthetic rehabilitation in children with ectodermal dysplasia is an important step toward improving their overall quality of life. The fixed prosthesis has the advantages of being more stable in the mouth with good child compliance and a good aesthetic outcome.

**Case presentation:**

Our patient was a 5-year-old Middle Eastern boy with oligodontia caused by ectodermal dysplasia. He was managed by fabrication of an upper functional space maintainer and a lower fixed partial denture to restore occlusion, masticatory function, aesthetics, and overall quality of life.

**Conclusions:**

The use of the fixed prosthesis in children is a new and evolving treatment modality that resolves many of the issues caused by removable prostheses. It accommodates jaw growth in the mandible, reduces the need to remake the prosthesis, and has an overall better aesthetic outcome.

## Background

Ectodermal dysplasia (ED) comprises a large group of rare genetic disorders affecting structures of ectodermal origin, such as skin, hair, nails, teeth, and sweat glands. More than 170 types are described in the literature [1], but the common ones are hypohidrotic and hidrotic, which differ in the degree of sweat gland function and hereditary pattern. Hypohidrotic ED is characterized by hypohidrosis due to the absence of or reduction in the number of sweat glands, hypotrichosis (sparse, light-colored scalp and body hair), and hypodontia [2]. Other prominent features include prominent forehead, flat bridge of the nose, and periorbital hyperpigmentation. The prevalence of ED ranges from approximately 1:10,000 to 1:100,000 worldwide [3], and it mostly affects males. Oral manifestations include partial or complete anodontia, abnormal shape of the teeth, enamel hypoplasia, reduced asymmetric alveolar ridge height, maxillary retrusion, and high palatal arch. Absence of teeth may cause masticatory difficulties, nutritional deficiencies, speech problems, and compromised facial appearance. Prosthetic rehabilitation in these patients is of utmost importance in order to achieve the desired functional, aesthetic, and psychological goals. Compared with traditional removable appliances, fixed prostheses are patient-friendly and provide a more stable and better hygienic and aesthetic result. Fixed prostheses improve speech and masticatory function and have fewer negative sequelae than other prosthetic replacements, which makes them the ideal option for young patients with multiple missing teeth [4].

## Case presentation

A 5-year-old Middle Eastern boy attended the pediatric dentistry department of the Dubai Health Authority with his parents with a chief complaint of multiple missing teeth. The child had been diagnosed with hypohidrotic ED since infancy by his attending pediatrician, but no other medical conditions were stated. The mother reported that the child experienced heat intolerance because of reduced sweating ability. The boy’s family history revealed that he is the third of five children; none of the other siblings have any medical conditions. The boy had a moderate build with a body mass index of 13.8 kg/m^2^.

**Fig. 1:**
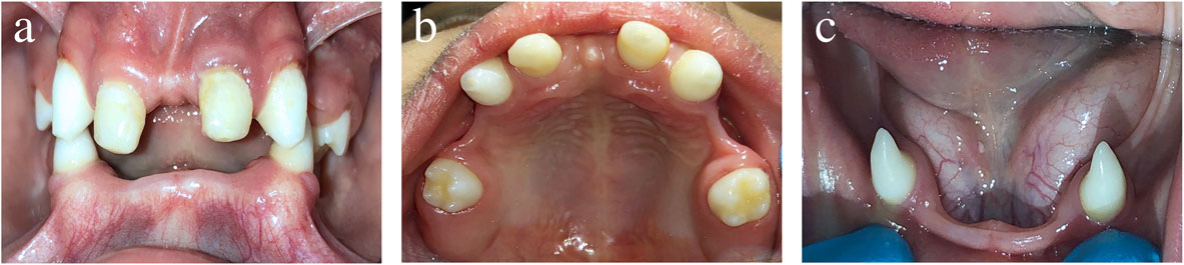
Preoperative intraoral views showing partial anodontia and thin atrophic alveolar ridges. **a** Frontal view. **b** Upper occlusal view. **c** Lower occlusal view

His extraoral examination revealed characteristics consistent with hypohidrotic ED: thin, sparse scalp hair; discoloration around the eyes; less eyebrow and eyelash hair; flat bridge of the nose; retrognathic maxilla; dry, thick lips; prominent chin; concave facial profile; and reduced lower facial height. The patient’s intraoral examination revealed that only eight teeth were present (upper primary second molars, upper permanent canines, upper primary canines, and lower permanent canines). His upper primary canines had a history of dental treatment with strip crowns to make them look like central incisors for aesthetic reasons. The patient exhibited a deep overbite and thin atrophic knife-edge alveolar ridges with loss of vestibular height, especially in the mandibular arch. The only occlusal contact was between the upper and lower permanent canines, with the canines being in class II. The patient’s oral mucosa was slightly dry, and his tongue was enlarged. He had an apparent reduced ability to produce saliva, and the saliva was viscous in nature. Some food debris and plaque accumulation were present, which was attributed to poor oral hygiene exacerbated by reduced cleansing ability of saliva (Fig. 1).

**Fig. 2:**
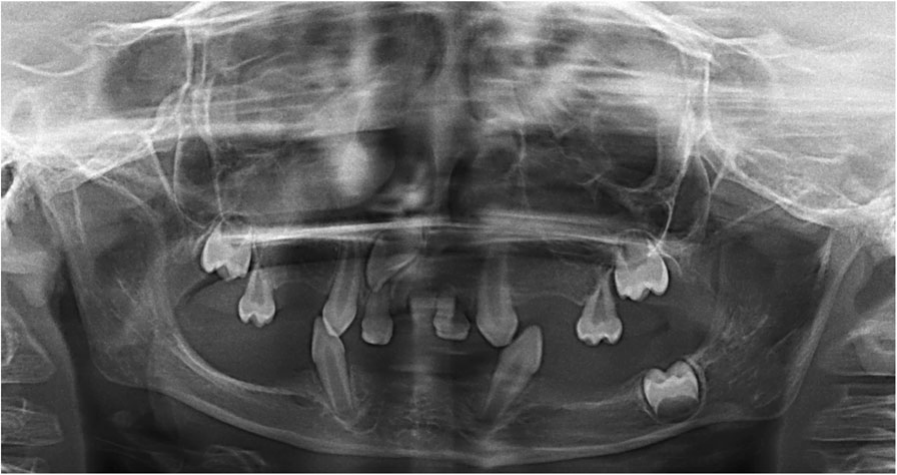
Panoramic radiograph showing the presence of four unerupted permanent tooth germs

Radiographic investigations included a panoramic radiograph that revealed the presence of four unerupted permanent tooth germs (upper right first molar, upper right lateral incisor, upper left first molar, and lower left first molar). The upper and lower permanent canines were erupted before the normal eruption time. Moreover, the radiograph revealed reduced height of the mandible, which is an expected finding in a patient with ED (Fig. 2).

Treatment options included removable partial dentures, overdentures, and fixed partial dentures (FPDs). Removable partial dentures are the most common treatment modality in children with oligodontia, but these prostheses can be less retentive and require frequent adjustments. For our patient, retention was significantly compromised by the thin atrophic nature of the alveolar ridges; therefore, removable partial dentures were excluded. Overdentures are more retentive than removable partial dentures, but they require elective pulp therapy of otherwise healthy abutment teeth. This option was excluded as well, owing to the parent’s request for a more conservative option. The last treatment option that was discussed with the family was FPDs, with the advantages of being more retentive and less demanding of the patient. The parents preferred a fixed prosthesis.

The proposed design for the upper arch was a Nance space maintainer with a saddle to replace upper primary first molars. A single incisor tooth was incorporated into the appliance to fill the big gap between the present anterior teeth to improve the aesthetics and smile. The plan was to use the appliance provisionally until the eruption of the upper right permanent lateral incisor. The lower arch was planned for an 8-unit ceramic bridge with ceramic-metal crowns on the two abutments (lower permanent canines) replacing the missing incisors and primary first molars. The lower appliance extended to the primary first molars without including the second primary molars to reduce the load on the abutment teeth. The pontic design was chosen to be of the modified ridge lap type, which has a concave fitting surface only at the facial surface, with the rest being convex, allowing it to contact the ridge only facially. Such a pontic design prevents food accumulation, making the appliance more hygienic. It also gives a better aesthetic result and is well tolerated by the ridge.

**Fig. 3:**
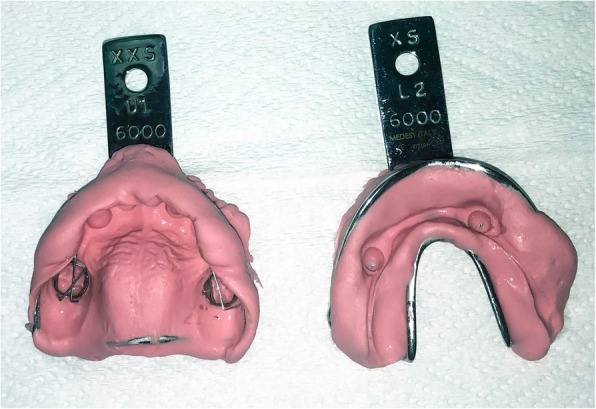
Upper and lower alginate impression used for the fabrication of the appliances

Upper and lower primary alginate impressions (perforated metal tray, alginate; 3M, St. Paul, MN, USA) (Fig. 3) and bite registration with wax were taken and sent to the laboratory. Casts were poured and analyzed to finalize the treatment plan. Occlusion was checked, and it was noted that there was a deep bite between the upper and lower permanent canines preventing the placement of acrylic teeth, so 2-mm occlusal reduction of the lower permanent canines was planned to open the bite and allow the coverage of the lower canines with metal-ceramic crowns.

At the patient’s next visit, the parents were informed about the procedure. The patient’s lower canines were carefully reduced using a high-speed diamond fissure bur to create a 2–3-mm free space between the upper and lower permanent canines. The patient’s teeth were smoothed with yellow stone, and topical fluoride varnish (5% sodium fluoride, White Vanish; 3M) was applied. The upper molar bands were fitted on upper primary second molars (size 27 Unitek orthodontic bands; 3M), and an alginate impression was taken to fabricate the upper Nance space maintainer with saddle, and a lower alginate impression was taken to fabricate the lower FPD. Shade selection was done with the VITA classical shade guide (VITA North America, Yorba Linda, CA, USA), and the shade A1 was chosen.

**Fig. 4:**
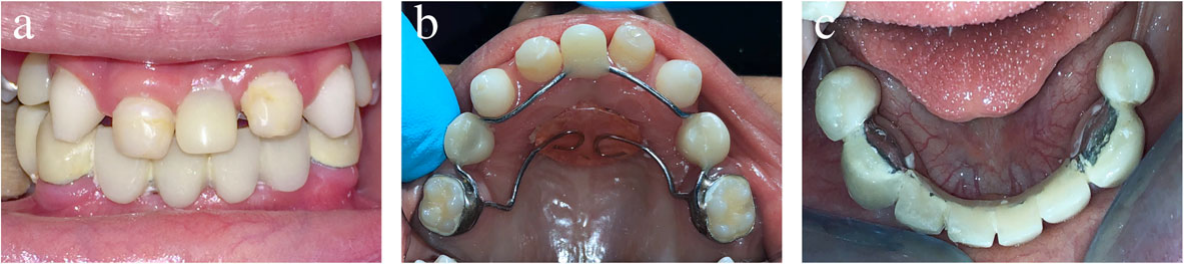
Postoperative intraoral views. **a** Frontal view. **b** Upper occlusal view of the upper Nance appliance with acrylic teeth. **c** Lower occlusal view of the lower fixed partial denture

At the patient’s next visit, the upper Nance appliance with acrylic teeth was tried in the patient’s mouth and cemented with ketac cement (3M ESPE Ketac-Cem; 3M) (Fig. 4b). We chose to deliver each appliance individually to gradually open the bite and train the patient in the new occlusion. Instructions were given to avoid consuming sticky foods, and oral hygiene instructions were provided. The boy’s parents were informed that he might experience some discomfort.

After 2 weeks, the boy was seen again in follow-up regarding the upper appliance and to deliver the lower appliance. The mother noted that the child felt comfortable with the appliance and was eager to replace his missing lower teeth. Upon intraoral examination, it was noted that gingival remodeling had occurred around the upper acrylic incisor with the formation of an interdental papilla between it and the adjacent natural teeth, giving the child a more natural aesthetic appearance. The lower FPD was tried in the mouth. Two retentive grooves were made on the cervical surface of the lower canines. Retention, resistance, aesthetics, phonetics, and occlusion were checked, and the appliance was cemented with ketac cement (Fig. 4c). Dietary and oral hygiene instructions were reinforced. Reestablishment of occlusion led to a favorable increase in vertical dimension with an increase in the height of the lower third of the face. The boy’s parents were instructed to encourage him to speak out loud to improve his phonetics. They were informed about the possibility of discomfort, difficulty eating, and unclear pronunciation in the first few days.

**Fig. 5:**
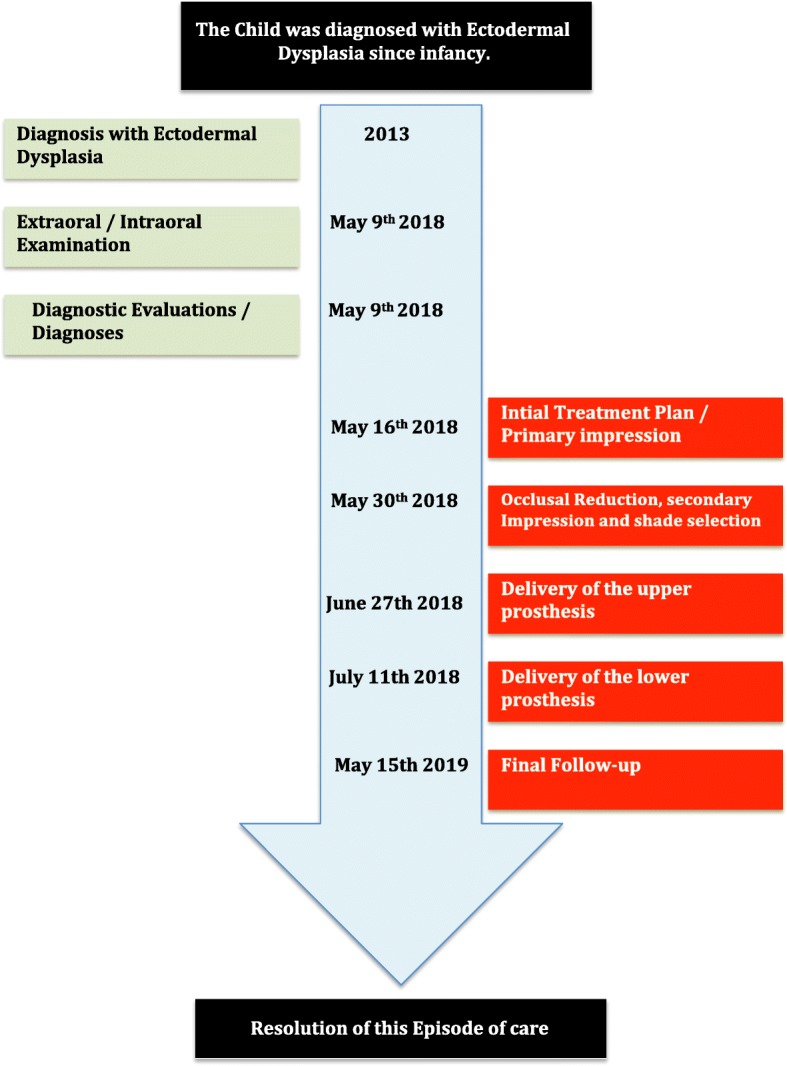
Timeline of the patient’s care

After 1 month of patient follow-up, the mother reported that the boy had slight difficulty in pronouncing some words, in addition to food accumulation around the lower appliance. The parents were reassured and were given speaking exercises for the child (counting, reading aloud) to help train his oral musculature to accommodate the new appliances. Oral hygiene instructions were reinforced, and the use of interdental brushes and superfloss to help clean around the acrylic teeth was demonstrated. Follow-up appointments were scheduled after 3 and 6 months. The appliances remained stable with no appreciable bone loss or gingival irritation. The parents reported that the patient had significant improvement in speech and masticatory function. Subsequent follow-up appointments were scheduled every 6 months. (A timeline of the patient’s care is shown in Fig. 5).

## Discussion

Dental management of a patient with ED is carried out using an interdisciplinary approach that requires the collaboration of the dental team to prevent, maintain, and restore the patient’s teeth, which improves aesthetics, phonetics, occlusion, masticatory function, and overall quality of life. As Nowak stated, “Treating the pediatric patient with ED requires the clinician to be knowledgeable in growth and development, behavioral management, techniques in the fabrication of a prosthesis, the modification of existing teeth utilizing various restorative techniques, the ability to motivate the patient and parent in the use of the prosthesis, and the long-term follow-up for the modification and/or replacement of the prosthesis” [5].

The first dental visit of patients with ED should occur as soon as the first tooth erupts, in order to establish the dental home [6] and to explain to the parents the treatment stages required as the child grows. Pigno *et al.* recommended use of a dental prosthesis before the child goes to school at around 3–4 years of age [7], which is in agreement with a systematic review by Schnabl *et al*., who found that the median age of prosthetic rehabilitation in patients with ED is 4 years [8]. Early prosthodontic rehabilitation of patients with ED helps to restore and normalize function of muscles of mastication and the skeletal growth pattern. Moreover, it helps to reduce the unwanted side effects caused by absence of teeth, such as alveolar ridge resorption, loss of vertical dimension, and a tendency to class III malocclusion [9]. It also helps to increase the child’s self-esteem and prevents any psychological trauma that may be caused by lack of teeth.

Prosthetic rehabilitation of patients with ED requires careful planning and knowledge to be able to design a prosthesis that accommodates their needs without having a negative effect on their quality of life. The most common treatment modality used for children with anodontia/hypodontia is a removable complete or partial denture, owing to ease of fabrication and modification in a growing child. However, retention and stability can be compromised because of the underdevelopment of alveolar ridges and the dryness of oral mucosa, which requires frequent relining or replacement, especially when a decreased vertical dimension of occlusion or abnormal mandibular posture is detected [10].

Overdentures provide a more retentive option and are used when teeth are present for support. They help preserve alveolar bone compared with complete dentures, as shown by Van Wass *et al.*, who found that there was a significant reduction in alveolar bone loss in the overdenture patient group after 2 years [11]. The only downside is that overdentures require aggressive tooth preparation and elective endodontic treatment of otherwise healthy teeth.

FPDs in children are gaining popularity because of their superior aesthetics, and improved retention and stability. Ou-Yang *et al.* [12] used FPDs with stainless steel bands cemented on primary molars to restore multiple missing teeth in two 3-year-old twin girls with ED. Their reasoning behind this appliance design was lack of anterior abutment teeth and flat alveolar ridges. They reported a favorable increase in occlusal vertical dimension and lip support leading to a superior aesthetic outcome. Furthermore, the band-retained partial dentures were well tolerated by the patients, and a positive improvement in speech was noted along with efficient increase in masticatory function, which made it easier to introduce solid foods into their diets. The dentures were removed on a monthly basis for cleaning purposes and were relined after 2 years following development of dental arches [12].

FPDs with rigid connectors are usually avoided in young, actively growing patients because they could prevent jaw growth, especially if the prosthesis crosses the midline [4]. However, transverse skeletal and alveolodental changes are less pronounced in the mandible than in the maxilla, and it is widely accepted that the lateral growth of the anterior mandible is completed by the age of 3 [13].

According to Barrow and White [14], intercanine width is established between the ages of 5 and 8 years. It is caused by distal migration of the primary canines into the primate spaces to accommodate the erupting permanent incisors. Because the lower permanent incisors were absent in our patient and the permanent canines were already erupted in the oral cavity, little intercanine growth was expected. Most growth will be occurring distal to the primary dental arches to accommodate the developing permanent teeth in an otherwise healthy patient, thus increasing arch length.

Regarding the upper appliance, a provisional Nance space maintainer with acrylic teeth to replace the missing primary first molars (D's) and close the big gap between the upper anteriors. The future options for replacing the missing anterior teeth in the maxilla can include a conventional bridge extending from canine to canine, a fiber-reinforced composite resin bridge, or modification of the design of the existing Nance appliance.

The mandibular arch was planned to receive an 8-unit ceramic bridge with ceramic-metal crowns on the two abutment teeth (lower permanent canines) replacing the missing incisors and primary first molars. According to Ante’s law, the total periodontal membrane area of the abutment teeth must equal or exceed that of the teeth to be replaced. Using the permanent lower canines as abutments to replace the four missing lower incisors is reasonable because of the long, wide roots of the canine and the small dimension of the mandibular incisor teeth, but the addition of two more pontics increases the load on the abutment teeth. Efforts were made to reduce the amount of load by ensuring the following:

1. The bridge was made as flat as possible in the mesiodistal aspect with minimum curvature in order to transport the forces favorably along the long axis of the abutment teeth.
2. Small premolar-sized teeth were chosen to replace the missing D’s with a small occlusal table.
3. The artificial lower D’s were opposed by artificial upper D’s, which exert less force than natural teeth.
4. The two abutment teeth were reinforced with metal coping.
5. A balanced occlusal scheme was chosen to distribute the occlusal load among all teeth.

The placement of dental implants in children is gaining popularity nowadays, especially in the anterior mandible, because the increase in the mandibular intercanine width ceases at a very young age. This provides an opportunity for dental implant placement without any negative influence on jaw growth. The National Foundation for Ectodermal Dysplasias recommends the placement of dental implants in the anterior mandible of children older than school age (7 years and older) [15]. However, the transverse growth of the maxilla continues until the age of 17 in boys when the midpalatine suture fuses, which contraindicates the use of maxillary dental implants in young patients [4]. Placement of dental implants in a growing patient carries the risk of growth cessation, implant submergence, or ankylosis. Furthermore, placement of a dental implant in patients with ED is challenging because of compromised bone quality, insufficient amount of bone, and continuous adjustments of the prosthesis. For those reasons, Cronin *et al.* concluded that implant therapy should start after the age of 15 years for girls and after age 18 years for boys to provide the best long-term prognosis with the minimum possible complications [16].

Periodic dental recall of patients with ED should be done at regular intervals to be able to monitor the patient’s growth and development and consequently adjust or replace the prosthesis accordingly. Vergo recommended relining/rebasing an intraoral prosthesis in a growing patient every 2–4 years and remaking a new prosthesis every 4–6 years [17]. Oral hygiene should be maintained by using a fluoridated dentifrice twice daily; a microbrush or superfloss should be used to clean around the artificial teeth; and topical fluoride varnish should be applied in the dental clinic.

## Conclusion

Prosthetic treatment of young patients with oligodontia caused by ED should be carried out using a multidisciplinary approach and should be tailored to every patient’s needs. Early replacement of missing teeth has a positive effect on growth and aids in restoring masticatory function, aesthetics, and speech and boosts the patient’s self-esteem, thus improving the patient’s overall quality of life.

## Data Availability

Data sharing is not applicable to this article, because no datasets were generated or analyzed during the p study.

## Abbreviations

ED: Ectodermal dysplasia
FPD: Fixed partial denture
